# Poor glycemic control and associated factors among patients with type 2 diabetes mellitus: a cross-sectional study

**DOI:** 10.1038/s41598-023-36675-3

**Published:** 2023-06-14

**Authors:** James J. Yahaya, Irene F. Doya, Emmanuel D. Morgan, Advera I. Ngaiza, Deogratius Bintabara

**Affiliations:** 1 Department of Pathology, School of Health Sciences, Soroti University, P. O. Box 211, Soroti, Uganda; 2 Department of Community Medicine, School of Medicine and Dentistry, University of Dodoma, Dodoma, Tanzania; 3 Deparment of Pathology, Muhimbili National Hospital, Dar-es-Salaam, Tanzania; 4 Department of Pathology, Muhimbili University of Health and Allied Sciences, Dar-es-Salaam, Tanzania

**Keywords:** Endocrinology, Medical research

## Abstract

Glycemic control is of paramount importance in care and management for patients with type 2 diabetes mellitus (T2DM). Poor glycemic control is a major health problem that greatly contributes to the development of diabetes related complications. This study aims to assess the prevalence of poor glycemic control and associated factors among outpatients with T2DM attending diabetes clinic at Amana Regional Referral Hospital in Dar-es-salaam, Tanzania from December 2021 to September 2022. A face to face interviewer semi-structured questionnaire was administered during data collection. Binary logistic regression under multivariable analysis was used to determine the independent predictors of poor glycemic control. A total of 248 patients with T2DM were included in the analysis with mean age of 59.8 ± 12.1 years. The mean fasting blood glucose was 166.9 ± 60.8 mg/dL. The prevalence of poor glycemic control was 66.1% (fasting blood glucose > 130 mg/dL or < 70 mg/dL). Failure to adhere to regular follow-up (AOR = 7.53, 95% CI = 2.34–19.73, p < 0.001) and alcoholism (AOR = 4.71, 95% CI = 1.08–20.59, p = 0.040) were the independent predictors of poor glycemic control. The prevalence of poor glycemic control observed in this study was significantly high. Emphasis should be placed on ensuring that patients have regular follow-up for their diabetes clinics and they should also continue modifying some of lifestyle behaviors including refraining from alcoholism, this can help them to have good glycemic control.

## Introduction

Diabetes mellitus (DM) is a metabolic syndrome which is characterized by hyperglycemia that develops as a result of lack or deficit of insulin secretion or sometimes resistance to its function^1^. The long-standing effects of hyperglycemia are associated with long-term damage, dysfunction, and failure of different organs, especially the eyes, kidneys, nerves, heart, and blood vessels^2,3^. DM is a significant public health problem of concern globally which contributes to approximately 5 million deaths annually from related complications^4,5^. It is estimated that, over 422 million adults are living with DM worldwide and this number is expected to increase to about 439 million adults by 2030 and 642 million by 2040^6^. The incidence of DM is increasing at a high rate globally, and approximately 80% of patients with DM reside in low-and middle-income countries (LMICs)^7^. Its negative impact is highest in resources-limited countries, where screening and access to care and treatment continue to be challenging for the fragile health systems available in place^8^.

Type 2 diabetes mellitus (T2DM) accounts for almost 90% of all forms of DM^9^. T2DM is a heterogeneous group of disorders which develops as a result of impaired secretion of insulin and/or resistance which both result into hyperglycemia^10^. The incidence of DM as well as T2DM vary from one country and another globally. In the United Kingdom for example, T2DM is the most common chronic disease and its incidence is increasing and between 2018–2019, it accounted for approximately 90% for adults with chronic diseases^11^. The incidence of T2DM is escalating globally including in most of LMICs particularly those which are found in the sub-Saharan Africa (SSA) region^12^. In 2018, Mayega et al. reported that the incidence of T2DM in Uganda was significantly increasing and over 400,000 people were living with T2DM^13^. The incidence of T2DM in Sudan was 20% in a study which was conducted in 2019^14^. Middle Eastern and North African (MENA) region, is one of the regions in the world with a large number of individuals living with DM and in 2019, approximately 54.8 million adults aged 20–79 were living with DM^15^. According to the International Diabetes Federation (IDF) report of 2022, it was reported that the prevalence of T2DM among adults was 10.3% and a total of 2,884,000 adults were living with DM^16^.

Achievement of good glycemic control among patients with T2DM is of paramount importance in delaying and/or preventing early onset of diabetes related complications which are associated with increased morbidity and mortality. Approximately, only 50% of diabetics worldwide achieve good glycemic control^12^ and in SSA, studies have shown majority of patients with T2DM have poor glycemic control. For example, in a study which was done in Ethiopia it was found that 74% of the patients with T2DM had poor glycemic control^17^. Also, in another study which was done in Uganda, the prevalence of poor glycemic control was found to be 84.3%^18^. In other two studies which were both done in Dar-es-salaam in 2014 reported prevalence of poor glycemic control among patients with T2DM of 69.7% and 64.4%^19,20^. In SSA, achieving good glycemic control as a result of proper management of diabetes faces many challenges including inadequate resources, lack of health priorities, inadequate funds for chronic disease management as well as low health insurance coverage and/or lack of health insurance^21^.

Both poor and good glycemic control in diabetics are usually predicted by different factors which may either modifiable or non-modifiable factors. For example, alcohol consumption which is a modifiable factor, is usually associated with failure of patients to achieving good glycemic control due to failure to exercise a number of beneficial actions including self-care and poor dietary intake, which both together contribute to poor glycemic control^22^. Studies have also reported a positive association of old age and male gender with poor glycemic and among diabetics despite contradicting findings in the literature^23,24^.

The problem of poor or inadequate ability of patients with T2DM to control their blood sugar level in Tanzania has not been fully investigated. This contributes to failure of realizing how big is the problem and this may also lead to lack of priority for allocation of funds for tackling the problem in order to improve the quality of life of the patients through reduction of morbidity and mortality. Therefore, the current study aims to assess the prevalence of poor glycemic control and associated factors among outpatients with T2DM attending diabetes clinic at a regional referral hospital.

## Methods

### Study design, setting, and duration

This was a cross-sectional analytical hospital-based study. The study was conducted at Amana regional referral hospital (ARRH) in Dar-es-salaam, Tanzania from December 2021 to September 2022. The hospital is located in Ilala municipal which is among the five municipals of the Dar-es-salaam city which is the largest business center of the country. The hospital provides treatment for approximately 43,800 patients per year of whom approximately 2080 usually attend the hospital for DM related treatments. Dar-es-salaam city is one of the highly populated cities in Tanzania with a significant number of adults who engage in substance use including alcoholism and smoking, which both are more likely to affect glycemic control for those with T2DM. In a recent study which was conducted in Dar-es-salaam, the reported prevalence of alcoholism and smoking was 31.3% and 6.3%, respectively^25^. Another study also reported prevalence of 17.2% and 8.7% among study subjects for alcoholism and smoking, respectively^26^.

### Patients’ characteristics and recruitment procedure

We included outpatients with T2DM who were attending diabetes clinic and they agreed to sign written informed consent. All patients with T2DM that were not willing to sign written informed consent and those who were seriously ill were excluded from the analysis.

### Sample size determination and sampling method

The sample size was calculated using the standard formula described by Kish Leslie considering prevalence for a single population: n = Z^2^ × p (100 − p)/e^2^^27^ assuming standard normal variables (z score) of 1.96 at 95% confidence interval, margin error (e) of 5% and prevalence (p) of 75.8% of poor glycemic control which was reported in the study by Rwegerera GM in Dar-es-salaam, Tanzania in 2014^20^. The total sample size obtained was 248 after considering attrition rate of 10%. Then the sample size of 248 was drawn from a study population of 480 subjects using lottery simple random sampling method. On average 20 patients could attended per week, out of whom up to 10 patients were sampled per week. Pieces of papers were cut and labeled “Yes” and “No” depending on the number of patients had attended. The pieces of papers were folded uniformly and placed in a container, which was covered tightly and shaken so as to mix them. Then the container was opened and every patient was asked to pick one piece of paper. Only those who picked a piece of paper labeled “Yes” were selected to participate in the study.

### Definition of variables

#### Physical activity

Physical activity was defined as any action involving body movement that results from relaxation and contraction of muscles that uses energy stores^28^. In this study, physical activity was measured as it was done in the previous study^29^. Patients who reported to do physical activity in less than 30 min per session per day were considered to have mild physical activity and those who reported to have physical activity in more than 30 min per session per day had moderate physical activity, and patients who reported to have physical activity for more than 45 min per session per day were regarded having vigorous activity.

#### Adherence to dietary intake

Diet for patients with T2DM was defined as the diet comprising of small and frequent more or equal to 5 per day that contains fruits, vegetables, high fibers and whole grains and low in sugars^30^. Adherence to dietary intake was measured using 10 items whose responses were grouped on Likert scale with scores ranging from 0 to 20 in which responses “always”, “sometimes” and “never” were scored 2, 1, and 0, respectively as it was done previously^31^. Good adherence to dietary intake was considered for scores from 15 to 20, 10 to 15 partial adherence and less than 10 scores were considered non-adherence to dietary intake.

#### Glycemic control

Good glycemic control was defined as an average of three consecutive fasting blood glucose measurements between 80 and 130 mg/dL. Poor glycemic control was defined as patients who had average blood glucose measurements on three consecutive visits > 130 or < 70 mg/dL^32^. Blood glucose level was measured using a glucometer (ONETOUCH Select Simple, serial number SAKQN77Z, Zug-Switzerland).

### Data collection and research instrument

Data were collected using a face to face interviewer administered semi-structured questionnaire which was adapted from a previous study particularly in measuring physical activity and adherence to dietary intake^31^. The questionnaire was divided into three parts: part 1 comprised sociodemographic characteristics (age of the patient, sex, education level, marital status, religion, residence, occupation, and income level), part II consisted of clinical characteristics (duration of T2DM, duration of treatment, route of medication, type of anti-diabetes, adherence to regular follow-up, complications, comorbidities, health insurance, and family history of T2DM, and part III included lifestyle behaviors (alcoholism, smoking, physical activity, and adherence to diabetes dietary intake).

Data collection was done by three researchers (I.F.D, J.J.Y and E.D.M). Validation of the adapted questionnaire was done by conducting a pre-test among 30 patients with T2DM from a different hospital within Ilala municipal in Dar-es-salaam. Data collection was done once per week when patients attended their diabetes clinic. Approximately, 10–20 min were spent during interview for every patient, and the process of interviewing patients was carried out in a secluded room for maintenance of privacy of the patients. Cronbach’s alpha of 0.75 for internal consistency of the validated questionnaire for evaluation of dietary adherence and physical activity was acceptable.

### Data analysis

Data were analyzed using SPSS software version 25.0. Descriptive statistics were used to summarize both categorical and continuous variables in frequencies and percentages and mean ± standard deviation (SD), respectively. Inferential statistics involved the use of binary logistic regression analysis in determining the predictors of poor glycemic control. All variables with p < 0.2 in univariate analysis were fitted into multivariable analysis for controlling confounding factors. Odds ratios (ORs) at 95% confidence interval (CI) were determined for assessing predictability of the independent variables. A two-tailed p < 0.05 was considered statistically significant.

### Consent to participate

Written informed consent was obtained from the patients and a copy has been kept by the corresponding author for future review by the Editor-In-Chief of the journal when requested.

### Ethics and consent

This study was approved by the Ethical Research Committee of the University of Dodoma (reference: MA.84/261/02 dated 3rd November 2021). We also confirm that all methods were performed in accordance with the relevant guidelines and regulations. All study participants signed informed consent and confidentiality was maintained by anonymizing identification of the participants. All study participants signed written informed consent and agreed their anonymized data to be published.

## Results

### Sociodemographic characteristics of the patients with T2DM

A total of 248 patients with T2DM were analyzed in the present study with response rate of 92.9%. The mean age of the patients was 59.8 ± 12.1 years (range 31–93 years). Majority 70.2% (174/248) of the patients were females and most 48.4% (120/248) of the patients were aged between 45 and 60 years. Over half 35.5% (88/248) of the patients had attained tertiary level of education and also over half 38.3% (98/248) were obese (Table 1).

**Table 1:** Sociodemographic characteristics of the study participants (N = 248).

Variables	Frequency (n)	Percentage (%)
Age (years)		
< 45	13	5.3
45–60	120	48.4
61–75	103	41.5
> 75	12	4.8
BMI (kg/m^2^)		
Normal (18.5–24.9)	66	26.6
Underweight (< 18.5)	7	2.8
Overweight (25–29.9)	80	32.3
Obese (≥ 30)	95	38.3
Sex		
Male	74	29.8
Female	174	70.2
Marital status		
Single	4	1.6
Married/cohabiting	152	61.3
Divorced/separated	15	6.0
Widower/widowed	77	31.1
Education level		
Informal	52	21.0
Primary	38	15.3
Secondary	70	28.2
Tertiary	88	35.5
Residence		
Urban	240	96.8
Rural	8	3.2
Religion		
Muslim	79	31.9
Christian	159	64.1
Others	10	4.0
Occupation		
Employed	72	29.0
Self-employed	79	31.9
Unemployed	97	39.1
Level of income (TZS) per month		
< 132,810	40	16.1
132,810–500,000	59	23.8
> 500,000	149	60.1

### Clinical characteristics of the patients with T2DM

The clinical characteristics of the patients are shown in Table 2. Majority 68.5% (170/248) of the patients had more than 5 years since the time they were diagnosed with T2DM and over half 56.5% (140/248) of the patients were on treatment between 5 and 10 years since they were put on anti-T2DM medication. Also, majority 63.3% (157/248) of the patients reported to have no regular follow-up of their DM clinics. Additionally, almost half 49.2% (122/248) of the patients had diabetic complications and retinopathy was the most common complication which consisted of 40.2% (49/122) of all complications.

**Table 2:** Clinical characteristics of the patients with T2DM (N = 248).

Variables	Frequency (n)	Percentage (%)
Family history of T2DM		
Yes	48	19.4
No	200	80.6
Health insurance		
Yes	179	72.2
No	69	27.8
Comorbidities		
Yes	154	62.1
No	94	37.9
Type of comorbidities		
Hypertension	137	55.2
HIV/AIDS	36	14.5
CKD	24	9.7
Others	11	4.4
Having diabetes complications		
Yes	122	49.2
No	126	50.8
Type of diabetes complications		
Nephropathy	21	17.2
Retinopathy	49	40.2
Neuropathy	13	10.7
Retinopathy + neuropathy	15	12.3
Neuropathy + nephropathy	24	19.7
Living condition		
Living alone	47	19.0
Supported by a family member	201	81.0
Adherence to regular follow-up		
Yes	91	36.7
No	157	63.3
Duration of T2DM since diagnosis (years)		
≤ 5	78	31.5
> 5	170	68.5
Route of anti-T2DM medication		
Oral	191	77.0
Intravenous	33	13.3
Mixed	24	9.7
Current medications used		
Metformin	204	82.3
Insulin	43	17.3
Gemer 1	1	0.4
Duration of treatment (years)		
< 5	43	17.3
5–10	140	56.5
> 10	65	26.2

Other comorbidities: peptic ulcer disease- 6 cases, arthritis-3 cases, and epilepsy-2 cases.

### Lifestyle behaviors of the patients with T2DM

Majority 79.8% (198/248) of the patients in this study reported not to have a tendency of taking physical activity in their history of being diabetic. Also, over one-third 33.1% (82/248) of the patients were not adhering to the guidelines concerning diabetic diets (Table 3).

**Table 3:** Lifestyle behaviors of the T2DM outpatients (N = 248).

Variables	Frequency (n)	Percentage (%)
Alcoholism		
Yes	36	14.5
No	212	85.5
Smoking		
Yes	7	2.8
No	241	97.2
Physical activity (per minutes per day)		
< 30 (mild)	21	8.5
30–45 (moderate)	22	8.9
> 45 (vigorous)	7	2.8
Inactivity	198	79.8
Dietary intake adherence		
Good adherence	100	40.3
Moderate adherence	66	26.6
No adherence	82	33.1

### Prevalence of poor glycemic control

**Figure 1:**
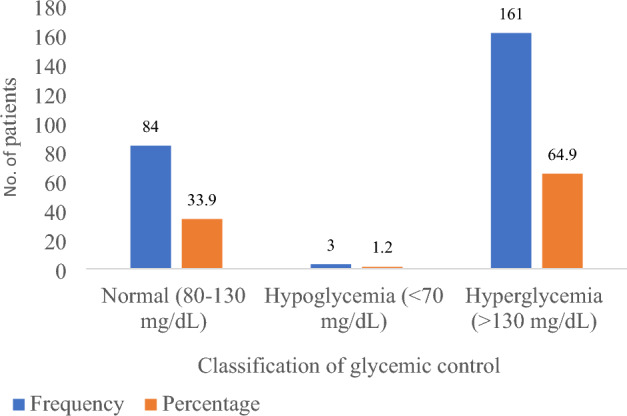
Prevalence of poor glycemic control among patients with T2DM (N = 248).

The statuses of glycemic control are shown in Fig. 1. The mean fasting blood glucose level was 166.9 ± 60.8 mg/dL (range 41.4–434.2 mg/dL). Majority 64.9% (161/248) of the patients had hyperglycemia and only 1.2% (3/248) had hypoglycemia, making a total of 66.1% (164/248) of patients with poor glycemic control in this study and the remaining 33.9% (84/248) patients had good glycemic control.

### Predictors of poor glycemic control

In multivariable logistic regression analysis; age, sex, level of education, health insurance, level of income, comorbidities, adherence to regular follow-up, duration of treatment, taking alcohol, and physical activity had p < 0.2 in univariate analysis and with no multicollinearity problems, and therefore were fitted in multivariable logistic regression analysis using enter method. Two independent variables remained significantly associated with poor glycemic control: failure of adherence to regular follow-up (AOR = 7.53, 95% CI = 2.34–19.73, p < 0.001) and taking alcohol (AOR = 4.71, 95% CI = 1.08–20.59, p = 0.040) (Table 4).

**Table 4:** Multivariable logistic regression analysis for determining predictors of poor for glycemic control.

Variables	Glycemic control		AOR (95% CI), p
	Poor: n (%)	Good: n (%)	
Age (years)			
< 60	78 (66.7)	39 (33.3)	1.00
≥ 60	86 (65.6)	45 (34.4)	2.23 (0.03–1.77), 0.160
Sex			
Male	49 (66.2)	25 (33.8)	1.41 (0.52–3.04), 0.862
Female	115 (66.1)	59 (33.9)	1.00
Education level			
No formal education	40 (76.9)	12 (23.1)	1.33 (0.32–5.56), 0.697
Primary	22 (57.9)	16 (42.1)	1.13 (0.32–4.02), 0.856
Secondary	43 (61.4)	27 (38.6)	1.08 (0.63–6.80), 0.223
Tertiary	49 (55.7)	39 (44.3)	1.00
Health insurance			
Insured	109 (60.9)	70 (39.1)	1.00
Not insured	55 (79.7)	14 (20.3)	1.50 (0.17–1.46), 0.303
Level of income (TZS/month)			
< 132,810	33 (82.5)	7 (17.5)	2.17 (0.70–8.45), 0.094
132,810–500,000	63 (61.8)	39 (38.2)	0.65 (0.07–3.22), 0.308
> 500,000	68 (64.2)	38 (35.8)	1.00
Comorbidities			
Yes	115 (72.8)	43 (27.2)	2.34 (0.92–5.97), 0.076
No	49 (54.4)	41 (45.6)	1.00
Adherence to regular follow-up			
Yes	17 (18.7)	74 (81.3)	1.00
No	147 (93.6)	10 (6.4)	7.53 (2.34–19.73), p < 0.001
Duration of treatment (years)			
< 5	58 (74.4)	20 (25.6)	1.32 (0.61–5.99), 0.102
5–10	43 (60.6)	28 (39.4)	0.40 (0.55–9.10), 0.083
> 10	63 (63.6)	36 (36.4)	1.00
Taking alcohol			
Yes	32 (88.9)	4 (11.1)	4.71 (1.08–20.59), 0.040
No	132 (62.3)	80 (37.7)	1.00
Physical activity			
Optimal	19 (65.5)	10 (34.5)	1.00
Mild	9 (42.9)	12 (57.1)	0.22 (0.04–1.16), 0.074
Inactive	136 (68.7)	62 (31.3)	1.57 (0.42–5.93), 0.504

Adjusted for: age, sex, education, health insurance, level of income, comorbidities, adherence to regular follow-up, alcoholism, physical activity, and duration of treatment.

## Discussion

This study aims to determine the prevalence of poor glycemic control and the factors that are significantly associated with increasing or decreasing levels of fasting blood glucose in a sample of Tanzanian patients with T2DM living in the Dar-es-salaam region. The findings have shown that, the vast majority of the patients that were assessed had poor glycemic control. It was also found that, failure to adhere to regular follow-up and alcoholism were the independent factors that was were significantly associated with poor glycemic control.

The vast majority of patients in this study had poor glycemic control. Similar high percentages of poor glycemic control of 69.7%, 64%, and 67.1% were reported in studies done Tanzania by Kamuhambwa et al.^19^, Hoffmeister et al.^33^, and Rwegerera^20^, respectively. Other studies done in Guinea and Cameroon^34^, Northern Ethiopia^17^, and South-Western Ethiopia^35^ reported poor glycemic control of 74%, 66.8%, and 72.7%, respectively, which are higher compared with the prevalence of poor glycemic control observed in the present study.

The prevalence of poor or suboptimal glycemic control among patients with T2DM from studies done in LMICs is higher compared with the one observed in most studies which have been reported in developing countries. For example, in the two studies which were done by Temma et al.^36^ and Matsumura et al.^37^ in Japan. The studies reported prevalence of poor glycemic control among patients with T2DM of 59.3% and 46.8%, respectively. These percentages of poor glycemic control are lower than that found in the present study and also other previous studies which were conducted in LMICs. In other studies which were done in Croatia^38^, Colombia^39^, and Thailand^40^ also reported relatively low prevalence of poor glycemic control of 58.8%, 52.7%, and 54.8%, respectively.

The discrepancy in prevalence of poor glycemic control among patients with T2DM reported in different areas may be based on different factors. Improved health systems including care for DM and good knowledge among patients and the general population regarding diseases such as DM observed among individuals in developed countries contribute to the improvement in control of blood sugar levels^41,42^. Fragile health systems associated with care of diabetes^43^, lack of political will to the engage the communities in improvement of health issues as well as lack of knowledge towards glycemic control among patients with diabetes in LMICs^44^; all account for the reported significantly high prevalence of poor glycemic control in most LMICs.

The difference in the tests used to measure glycemic control could also explain the variation in the prevalence of glycemic control. For example, use of fasting blood glucose test to measure blood glucose levels is more likely to give different results from results which may be produced when glycated haemoglobin (HbA1c) test if used. Also, it has been reported that, the use of different cut-off points when using HbA1c test in measuring blood glucose level may lead to the variation in prevalence of poor glycemic control. For instance, some studies considered HbA1c ≥ 7%, 8% and 7% as cut-off points for patients to be considered to have poor glycemic control^45^.

Regarding factors associated with poor glycemic control in this study, we observed that lack of adherence to regular follow-up among patients was significantly associated with poor glycemic control. Patients who had irregular follow-up were almost 8 times more likely to have poor glycemic control compared to patients with adherence to regular follow-up. This is similar to the findings in the studies of Zhao et al.^46^ and Asao et al.^47^ in which it was also observed that, patients with diabetes who had optimal number of hospital visits for DM clinics was associated with good glycemic control and good quality of life. Furthermore, Yigazu et al.^48^ and Ramirez et al.^49^ also reported that, patients with T2DM who had regular follow-up had good glycemic control compared to patients that did not have regular follow-up.

The benefits of having regular follow-up among patients with DM depend on the condition of the patient. For patients with controlled blood sugar, the frequency of follow-up does not help in further improving the clinical outcomes of the patients^50^. However, patients with T2DM whose glycemic control is suboptimal or poor, are more likely to improve when they maintain regular follow-up for their diabetes clinics over a period of time^51^. Therefore, it is important for patients with diabetes to have regular follow-up during diabetes clinics.

In this study, taking alcohol was associated with high odds for poor glycemic control. The odds for poor glycemic control among patients who were taking alcohol were significantly higher than in patients who were non-alcoholic. Other studies have also reported a positive association between poor glycemic control with alcohol intake among diabetics. Salama et al. and Abdissa et al. reported that alcohol intake was associated with poor glycemic control^52,53^. Alcohol intake is detrimental particularly for vulnerable people such as those with diabetes, which usually affects the ability of the patients to practice self-care for themselves as well as affecting vital body organs^54^. Studies have shown that, excess alcohol intake, particularly in patients with DM can lead to accumulation of certain acids including acetic acid and acetaldehyde in the blood circulation which leads to lethal complications including damage to the organs, dehydration, and increased blood pressure^54–56^. Additionally, alcohol intake can worsen diabetes-related medical complications, such as disturbances in fat metabolism, nerve damage, and eye disease^53,55^.

Alcohol consumption for patients with DM is associated with decreased food intake^57^ and it is also linked with reduced desire to adhere to dietary guidelines^58^, which both are more likely to impair glycemic control. Furthermore, it has also been shown that alcohol interferes with attention to diet and medication due to impaired ability to make decisions^53,58^. This therefore, affects self-care related lifestyle behaviors including physical activity exercise as well as glucose self-monitoring. Other factors for example age of the patients (≥ 60 years), duration of treatment (< 5 years), having comorbidities, low family income, having informal education, and not having health insurance even though had increased risk for poor glycemic control, but they were not statistically significant.

The findings from this study have shown that, the problem of poor glycemic control among T2DM patients in Tanzania which is among developing countries is still a major health problem which needs collective majors and holistic approach among stakeholders in mitigating its adverse effects. Large multicenter epidemiologic studies in future may help to provide comprehensive and detailed explanations predictors and other related contributing factors.

### Study limitations

The study had some limitations including the following: knowing the fact that our data involved patients only from a single health facility in the country, therefore, the findings cannot be generalized. The observed high prevalence of poor glycemic control in this study may be exaggeration of the actual true picture due the fact that data were collected from patients attending at a hospital, this might have resulted into selection bias. Our study cannot establish the causal-effect relationship due to the fact that it is cross-sectional. We could not collect information on drug prescription due to inconsistent documentation which we observed. Also, we could not involve use of HbA1c in determining glycemic control due to financial constraints.

## Conclusion

This study reports a significant high prevalence of poor glycemic control among patients with T2DM. This is because for every ten patients, approximately seven of them had poor glycemic control. Alcohol intake and failure to adhere to regular follow-up among patients were the independent factors that were significantly associated with poor glycemic control. This shows that, positive modification of the lifestyle behaviors of patients with T2DM in Tanzania would help to improve glycemic control and prevent development of related complications.

## Data Availability

The dataset used for this study is restricted by the Research Ethical Committee of the institution detail due to containing sensitive patient information, however, it can be accessed upon reasonable request from the Directorate of Research Publication, and Consultancy (DRPC), University of Dodoma, P. O. Box 259, Dodoma, Tanzania. Email: drpc@udom.ac.tz.

## Abbreviations

T2DM: Type 2 diabetes mellitus
DM: Diabetes mellitus
NCDs: Non-communicable diseases
LMICs: Low-and middle-income countries
CKD: Chronic kidney disease
SSA: Sub-Saharan Africa
